# Significant Reduction of Antibiotic Consumption and Patients’ Costs after an Action Plan in China, 2010–2014

**DOI:** 10.1371/journal.pone.0118868

**Published:** 2015-03-13

**Authors:** Lidao Bao, Rui Peng, Yi Wang, Ruilian Ma, Xianhua Ren, Wenbin Meng, Fusheng Sun, Junxia Fang, Ping Chen, Yang Wang, Qiuhong Chen, Jian Cai, Jian Jin, Jinhui Guo, Shucheng Yang, Xiaomei Mo, Enjing Zhang, Yuerong Zhang, Zhaoxin Lu, Binbin Chen, Xiujuan Yue, Meijun Zhu, Yingying Wang, Xinchao Li, Yuan Bian, Shaoshan Kong, Wenfei Pan, Qian Ding, Jun Cao, Rupin Liu, Nan Chen, Xuelian Huang, Agula B, Haijun Lyu

**Affiliations:** 1 Department of Pharmacy, Affiliated Hospital of Inner Mongolia Medical University, Hohhot, Inner Mongolia, China; 2 Department of Obstetrics, Affiliated Hospital of Inner Mongolia Medical University, Hohhot, Inner Mongolia, China; 3 Department of Pharmacy, People’s Hospital, Qingdao, Shandong, China; 4 Department of Pharmacy, The Third People’s Hospital, Fuyang, Zhejiang, China; 5 Department of Pharmacy, Central Hospital, Zibo, Shandong, China; 6 Department of Pharmacy, Women and Children’s Hospital, Maanshan, Henan, China; 7 Department of Pharmacy, The 175th Military Hospital of the People’s Liberation Army, Zhangzhou, Fujian, China; 8 Department of Pharmacy, Fengxian District Psychiatric Center, Shanghai, China; 9 Department of Pharmacy, The Ninth Affiliated People’s Hospital of Shanghai Transport University, Shanghai, China; 10 Department of Pharmacy, Tuberculosis Hospital, Zhengzhou, Henan, China; 11 Department of Pharmacy, Women and Children’s Hospital, Taiyuan, Shanxi, China; 12 Department of Pharmacy, Women and Children’s Hospital, Qingdao, Shandong, China; 13 Department of Pharmacy, The Third Hospital, Wuhan, Hubei, China; 14 Department of Pharmacy, Huli District Women and Children’s Hospital, Xiamen, Fujian, China; 15 Department of Emergency, Haici Medical Group, Qingdao, Shandong, China; 16 Department of Pharmacy, Psychiatric Center, Xiamen, Fujian, China; 17 Department of Pharmacy, Beijing Youan Hospital of Capital Medical University, Beijing, China; 18 Department of Pharmacy, Minhang District Psychiatric Center, Shanghai, China; 19 Department of Pharmacy, Dongchangfu District Women and Children’s Hospital, Liaocheng, Shandong, China; 20 Department of Pharmacy, Lintong District Chinese Medicine Hospital, Xian, Shanxi, China; 21 Department of Pharmacy, People’s Hospital, Chengdu, Sichuan, China; 22 Department of Pharmacy, Women and Children’s Hospital, Zhuhai, Guangdong, China; 23 Department of Pharmacy, Buwai Cardiology Hospital, Beijing, China; 24 Department of Pharmacy, Affiliated Children’s Hospital of Capital Pediatrics Research Center, Beijing, China; 25 Department of Pharmacy, The Fifth People’s Hospital, Suzhou, Zhejiang, China; 26 Department of Pharmacy, Central Hospital, Xinyang, Henan, China; 27 Department of Pharmacy, People’s Hospital Affiliated to Southern China Medical University, Zhengzhou, Henan, China; 28 Department of Pharmacy, People’s Hospital, Luo County, Guangdong, China; 29 Department of Mongolian Medicine, Inner Mongolia Medical University, Hohhot, Inner Mongolia, China; 30 Scientific Research Center, Affiliated Hospital of Inner Mongolia Medical University, Hohhot, Inner Mongolia, China; NERC Centre for Ecology & Hydrology, UNITED KINGDOM

## Abstract

**Introduction:**

On July 1, 2011, the Chinese government launched a national Action Plan for antibiotic stewardship targeting antibiotic misuse in public hospitals. The aim of this study was to evaluate the impacts of the Action Plan in terms of frequency and intensity of antibiotic utilization and patients costs in public general hospitals.

**Methods:**

Administrative pharmacy data from July 2010 to June 2014 were sampled from 65 public general hospitals and divided into three segments: (1) July 2010 to June 2011 as the preparation period; (2) July 2011 to June 2012 as the intervention period; and (3) July 2012 to June 2014 as the assessment period. The outcome measures included (1) antibiotic prescribing rates; (2) intensity of antibiotic consumption; (3) patients costs; and (4) duration of peri-operative antibiotic treatment in clean surgeries of thyroidectomy, breast, hernia, and orthopedic procedures. Longitudinal and cross-sectional analyses were conducted.

**Results:**

Longitudinal analyses showed significant trend changes in the frequency and intensity of antibiotic consumption, the patients’ costs on antibiotics, and the duration of antibiotic treatment received by surgical patients undergoing the 4 clean procedures during the intervention period. Cross-sectional analyses showed that the antibiotic prescribing rates were reduced to 35.3% and 12.9% in inpatient and outpatient settings, that the intensity of antibiotic consumption was reduced to 35.9 DDD/100 bed-days, that patients’ costs on antibiotics were reduced significantly, and that the duration of peri-operative antibiotic treatment received by surgical patients undergoing the 4 types of clean procedures decreased to less than 24 hour during the assessment period.

**Conclusion:**

The Action Plan, as a combination of managerial and professional strategies, was effective in reducing the frequency and intensity of antibiotic consumption, patients’ costs on antibiotics, and the duration of peri-operative antibiotic treatment in the 4 clean surgeries.

## Introduction

The increasing prevalence of antibiotic resistance has been recognized as a public health threat worldwide, especially in China [1]. The prevalence of methicillin-resistant *Staphylococcus aureus* (MRSA), extended-spectrum β-lactamase-producing *Escherichia coli*, imipenem-resistant *Pseudomonas aeruginosa*, and imipenem-resistant *Acinetobacter baumannii* accounted for 50% to 70% of nosocomial infections in China in 2010 [2]. Antibiotic overuse and misuse are major contributing factors for antibiotic resistance [3,4]. An epidemiological research found that antibiotics were prescribed for 68.9% of hospitalized patients, among whom 37.0% of the patients were subject to a combination of multiple antibiotics treatment, and that the average intensity of antibiotic consumption was 80.1 Defined Daily Doses/per 100 patient-days (more than twice of the average value worldwide) in China in 2010 [5].

According to recommendations from the World Health Organization (WHO), healthcare authorities should put emphasis on four aspects to contain antibiotic resistance, including (1) establishment of a national system to address antibiotic resistance; (2) legislation followed by enforcement to prevent illegal sales or marketing of prescription drugs targeting microbial infections; (3) education and stewardship on rational antibiotic use; and (4) strict adherence to guidelines aiming to prevent infections in the entire healthcare facilities [6]. Collectively, WHO suggests that antibiotic resistance should be addressed by administrative strategies together with professional strategies at national, regional, and institutional levels. Implementation and enforcement of a holistic antibiotic stewardship program have shown positive effects in combating antibiotic resistant pathogens, as was reported in a recent meta-analysis of 89 studies [7]. On July 1, 2011, the Chinese government initiated a national public health campaign targeting antibiotic resistance [8]. The campaign was a national antibiotic stewardship action plan (hereafter referred to as the Action Plan) in a series of government regulations over the past decade by incorporating administrative and professional strategies into an overall antibiotic stewardship program. Since its inception, the rampant antibiotic consumption driven by multifaceted factors have been reduced as a result of enforced antibiotic management and optimized antibiotic prescribing in China [1,9]. However, there are few researches evaluating the overall impact of the Action Plan due to practical and methodological challenges.

The aim of this Research Article was to bridge the gap by examining the pharmacoeconomic impacts of the Action Plan. Administrative pharmacy data and electronic medical records were extracted from the hospital information systems in 65 public general hospitals across China. The impacts of the Action Plan on antibiotic consumption and economic costs were analyzed using segmented regression analysis of interrupted time series (ITS).

## Methods

### Policy interventions by the Action Plan

The Action Plan for combating antibiotic resistance officially took into effect on July 1, 2011 [8]. Since compulsory participations from secondary and tertiary public hospitals (corresponding to Class II and III hospitals according to China’s hospital classification system) were dictated by the Ministry of Health, a separate control group characterized by a randomized control trial was not feasible. Therefore, we decided to evaluate the impact of the Action Plan using segmented regression analysis of ITS as well as cross-sectional analyses to provide a whole picture. Before the initiation of the Action Plan, 3 guidelines that had effects on antibiotic prescribing had been enacted by the Ministry of Health: (1) *Regulations on Prescription Management* issued on February 10, 2010, which highlights pharmacy validation by clinical pharmacists on medication use, especially on antibiotic prescribing [10]; (2) *Regulations on pharmacy administration in medical institutions* issued on January 30, 2011, which underscores the key roles of Drug and Therapeutics Committees (DTCs) and clinical pharmacists in formulary control, concurrent prescribing monitoring, and pharmacy validation [11]; and (3) *Action Plan for Antibiotic Stewardship in Clinical Application* issued on April 25, 2011, which outlines the key elements in developing and implementing a framework for antibiotic stewardship in public hospitals [8]. In our study, the 3 major policy changes mentioned above were considered as a preparation phase prior to the official initiation of the Action Plan started on July 1, 2011.

The Action Plan dictated: (1) that the chief administrators (leaders groups) of the hospital should be responsible for patient outcomes and cost-effectiveness of antibiotic utilization; (2) that the prescription evaluation system should be enforced with manifested disciplinary actions against infringement [10]; (3) that a functional infectious disease department and microbiology department should be enforced to work in collaboration with clinical pharmacists and other professionals with routine responsibilities of infection control; and (4) that a structured (non-restricted, restricted, and very-restricted) antibiotic use policies should be established and clinicians should be trained and accredited before being assigned the level-of-use antibiotic prescribing or dispensing privileges. Non-restricted (first-line) antibiotics refer to those with proven efficacy, relatively low price and little effect on antibiotic resistance; restricted antibiotics (second-line) refer to those with proven efficacy, relatively high price and greater effect on antibiotic resistance; and very-restricted antibiotics refer to those with known adverse effects and a tendency to cause antibiotic resistance. As a managerial tool, specific goals were set by the Action Plan: (1) the proportion of patients receiving antibiotic prescription (hereafter referred to as antibiotic prescribing rates) should be less than 60% for hospitalized patients or less than 20% for outpatients; (2) the intensity of antibiotic consumption should be less than 40 DDD/100 bed-days; (3) the proportion of patients receiving antibiotic prophylaxis for clean surgeries should be less than 30%; and (4) antibiotic prophylaxis should be administered 30 to 120 minute prior to surgical incision and the duration of the prophylaxis should not extend beyond 24 hour.

Apart from regular dissemination sessions through routine DTC meetings in respective hospitals, the Ministry of Health regularly held national education programs for physicians and managerial personnel. Supervision on the compliance with the Action Plan was conducted twice a year by the healthcare authorities. These measures are an ongoing process, so is the refinement of the guidelines for prescribing antibiotics. On May 8, 2012, the Chinese MoH issued a revised version of antibiotic guidelines entitled *Regulations on Clinical Applications of Antibiotics* [12], aiming to improve rational antibiotic utilization in all medical units with detailed listings of indications, contraindications, dose, form, route, frequency and duration of administration on antibiotics in the formulary of public hospitals.

### Study design

Conceived by the first author in May 2014, a research on the pharmacoeconomic impacts of the Action Plan was discussed by peers on a professional clinical pharmacist community (http://www.clinphar.cn/). The inclusion criteria required the completeness and availability of administrative pharmacy data and electronic medical records from all medical units, excluding the departments of pediatrics and hematology, over the past 4 years (July 2010 to June 2014). By stratified sampling based on classification (i.e., secondary or tertiary) of public general hospitals, the research protocols were sent to the pharmacy departments of 300 public general hospitals. Among the 152 hospitals that responded, 65 hospitals (30 tertiary hospitals and 35 secondary hospitals) were selected depending on the inclusion criteria. The stratification of sampling was based on the ratio of the total number of medical encounters in tertiary hospitals to the number in secondary hospitals in 2011 (898 million in tertiary hospitals vs. 992 million in secondary hospitals) [13]. This research was approved by the Ethics Committee of the Affiliated Hospital of Inner Mongolia Medical University. The Ethics Committees of the 65 hospitals approved this research on condition of not being identified.

Since 3 major policy changes mentioned above had occurred before the initiation of the Action Plan on July 1, 2011, we chose data points of 12 months prior to the initiation. The coauthors of our study agreed to identify this time period as a preparation period. Further, it was reasonable to postulate that there were lagged effects of the Action Plan as disseminating and implementing the Action Plan occurred during an extended period of time. After preliminary data analysis on the collected data, the study period was divided into 3 segments characteristic of the segmented regression analysis, including Segment 1: the preparation period (July 2010 to June 2011); Segment 2: the policy intervention period (July 2011 to June 2012); and Segment 3: the assessment period (July 2012 to June 2014).

### Outcome measures and data collection

The outcome measures were the managerial goals set by the Action Plan, as described above. Antibiotic consumption data were collected from the administrative pharmacy data and electronic medical records in the 65 hospitals using a self-developed program, by which the consumed medication including antibiotics (ATC code: J01–J05) were converted into the ATC/DDD system (WHO, version 2013) [14]. Antibiotic prescribing rates were calculated for inpatient, outpatient, and emergency settings in the 65 hospitals. The data of the antibiotic consumption were normalized to DDD/100 bed-days for inpatients, and to DDD/1000 outpatient-days for outpatients. Inpatients’ costs (including the costs on hospital study, medication, antibiotics, and very-restricted antibiotics) were extracted from inpatients’ discharge records. Outpatients’ costs on medication, antibiotics, and very-restricted antibiotics were collected from hospital information systems. The patients’ costs were then converted into U.S. dollars (1 U.S. dollar = 6.15 CNY). The average duration of hospital stay as well as peri-operative antibiotic treatment received by patients undergoing 4 types of clean surgeries (thyroidectomy, breast surgery, hernia, or orthopedic procedures) were extracted. Finally, the collected data were aggregated as evenly-spaced average monthly data characteristic of time series data points.

### Statistical analysis

Segmented regression analysis of ITS was conducted to analyze the aggregated monthly data of interest from indicated hospitals. The data points were then divided into 3 segments corresponding to the preparation period, policy intervention period, and assessment period. Two parameters define each segment of a time series: (1) level, which is the value of a time series at a beginning of a given times series (e.g., the *y*-intercept); and (2) trend, which is the rate of change of an outcome measure of interest (e.g., the slope). Segmented regression analysis of interrupted time series allows us to assess, in statistical terms, how much an intervention changed an outcome of interest, immediately and over time; instantly or with delay; transiently or long-term [15]. Model 1 below can be used to evaluate the changes in level and trend caused by a single intervention. Model 2 below can be used to evaluate the changes in time series with more than one change points which are characterized by lagged effects and/or interventions that occur over an extended period of time [15]. Given the lagged effects revealed by preliminary analysis and multiple interventions occurred during the policy intervention period, Model 2 was the statistical model used in this study:

$$Y_{t}=\;\beta_{0}+\;\beta_{1}\times time_{t}+\;\beta_{2}\times intervention_{t}+\;\beta_{3}\times time\;after\;intervention_{t}+e_{t}$$ (Model1)

$$\begin{array}{l} Y_{t}=\;\beta_{0}+\;\beta_{1}\times time_{t}{}_{}+\;\beta_{2}\times intervention_{t}+\;\beta_{3}\times time\;after\;intervention_{t}\\ +\;\beta_{4}\times assessment_{t}{}_{}+\;\beta_{5}\times time\;after\;assessment_{t}+e_{t} \end{array}$$ (Model2)

In Model 2, β_0_ estimates the level of the outcome during the preparation period; β_1_ estimates the trend during the preparation period; β_2_ estimates the level change during the policy intervention period; β_3_ estimates the trend change during the policy intervention period; β_4_ estimates the level change during the assessment period; β*5* estimates the trend change during the assessment period. *Yt* is the average monthly value of the outcome measure of interest at month *t*; *time* is a continuous variable indicating time in months at time *t* starting from the preparation period (time 0); *intervention* is an indicator for time *t* occurring before (*intervention* = 0) or after (*intervention* = 1) the policy intervention which was implemented at month 13 (e.g., July 2011); *time after intervention* is a continuous variable counting the month after the policy intervention at time *t*, coded 0 before the policy intervention or 1–36 after the policy intervention; *assessment* is an indicator for time *t* occurring before (*assessment* = 0) or after the assessment (*assessment* = 1) which was implemented at month 25; *time after assessment* is a continuous variable counting the months after the assessment at time *t*, coded 0 before the assessment or 1–24 after the assessment. The last term in Model 2 is the error term *e*_*t*_, representing variation unexplained by the segmented regression model. The error term *e*_*t*_ is assumed to be comprised of a Gaussian random error (white noise) and an potential error term at time *t* that may be correlated to errors (the autoregressive component) or shocks (the moving-average component) at preceding or subsequent time points [15]. To avoid overestimating the significance of effect of the policy intervention, the error term *e*_*t*_ is reduced to a white noise by fitting an ARMA model to the error term *e*_*t*_ [15].

Statistics were analyzed using Statistics Analysis System (SAS; version 9.2). The effects of policy intervention are expressed as changes in level and trend and their corresponding standard errors. Segmented Regression analysis of ITS was conducted using PROC ARIMA of SAS. In addition, the impacts of the Action Plan were evaluated by whether the managerial goals on antibiotic consumption had been achieved during the assessment period; therefore, cross-sectional analyses were conducted on the average yearly values of outcome measures during the 3 periods using PROC MIXED of SAS. Bar charts were plotted for data visualization, with error bars representing standard deviations. Normality was checked by Shapiro-Wilk test, and homoscedasticity was checked using plots of residuals against the predicted values. The significance level was 0.05.

## Results

### Changes in antibiotic prescribing rates and intensity of antibiotic consumption

**Fig 1:**
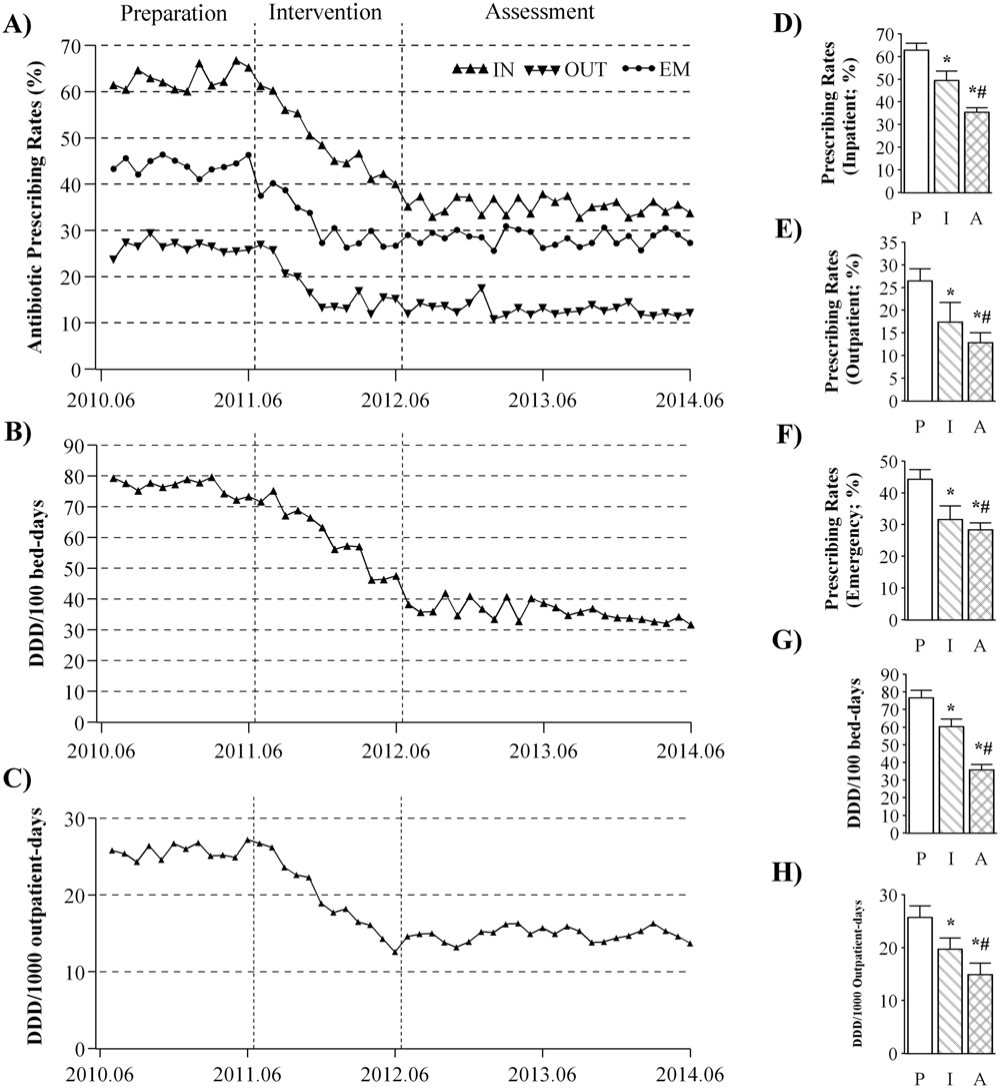
Changes in antibiotic prescribing rates and intensity of antibiotic consumption. Time series of average monthly value of the antibiotic prescribing rates (A) were plotted for inpatient (IN), outpatient (OUT), and emergency (EM) settings. The data on intensity of antibiotic consumption were plotted for the inpatient (B) and outpatient settings (C). Cross-sectional analyses were conducted by comparing the average yearly data on antibiotic prescribing rates in inpatient (D), outpatient (E) and emergency (F) settings as well as the intensity of consumption in the inpatient (G) and outpatient (H) settings. P: preparation; I: intervention; A: assessment; *significant difference in intervention/assessment vs. preparation; ^#^significant difference in assessment vs. intervention.

The impacts of the Action Plan on antibiotic consumption in the 65 general hospitals are shown in Fig. 1. The changes of level and trend with respects to antibiotic prescribing rates during the preparation, intervention, and assessment periods are summarized in Table 1. Fig. 1A shows that the average antibiotic prescribing rates declined by 2.27% (*se* = 0.22; *p* < 0.001), by 1.07% (*se* = 0.37; *p* = 0.004), and by 1.31% (*se* = 0.19; *p* < 0.001) per month in the inpatient, outpatient and emergency settings during the intervention period, respectively (Table 1). Cross-sectional analyses showed that the average prescribing rate was reduced significantly from 62.9% during the preparation period to 35.3% during the assessment period in the inpatient settings (*p* < 0.001; Fig. 1D). Similar reductions in prescribing rates were observed in both outpatient (26.4% vs. 12.9%; *p* < 0.001) and emergency (44.2% vs. 28.4%; *p* < 0.001) settings during the assessment period compared with the preparation period (Fig. 1E and 1F).

**Table 1:** Regression analyses of ITS on the changes in antibiotic consumption.

	β1-trend (preparation)	β2-level change (intervention)	β3-trend change (intervention)	β4-level change (assessment)	β5-trend change (assessment)
**RATE_I**	0.30 (0.16)	-2.32 (1.55)	-2.27 (0.22)***	-2.67 (1.30)*	1.93 (0.17) ***
**RATE_O**	-0.08 (0.24)	0.43 (1.99)	-1.07 (0.37) **	1.16 (1.79)	1.13 (0.27) ***
**RATE_E**	0.10 (0.15)	-5.58 (1.60) ***	-1.31 (0.19) ***	4.25 (1.17) ***	1.17 (0.14) ***
**DDD_I**	-0.39 (0.21)	2.84 (2.13)	-2.23 (0.30) ***	-7.06 (1.74) ***	2.39 (0.22) ***
**DDD_O**	0.06 (0.10)	1.67 (0.88)	-1.31 (0.14) ***	2.03 (0.75)**	1.25 (0.10) ***

Changes in level and slope in antibiotic prescribing rates in inpatient (RATE_I), outpatient (RATE_O) and emergency (RATE_E) settings in the 65 general hospitals were analyzed using segmented regression analysis of ITS. The intensity of antibiotic consumption was expressed as DDD/100 bed-days for inpatient settings (DDD_I) or DDD/1000 outpatient-days for outpatient settings (DDD_O). The parameters of β1 to β5, expressed as Mean (SE), were described in the Methods.

*p < 0.05;

**p < 0.01;

***p < 0.001.

Fig. 1B and 1C show that the average intensity of antibiotic consumption decreased per month by 2.23 DDD/100 bed-days (*se* = 0.30; *p* < 0.001) in the inpatient settings, and by 1.31 DDD/1000 outpatient-days (*se* = 0.14; *p* < 0.001) in the outpatient settings during the intervention period (Table 1). Cross-sectional analyses showed that the average intensity of inpatients’ antibiotic consumption dropped from 76.6 DDD/100 bed-days during the preparation period to 35.9 DDD/100 bed-days during the assessment period (*p* < 0.001; Fig. 1G), and that the intensity of outpatients’ antibiotic consumption declined from 25.7 DDD/1000 outpatient-days during the preparation period to 14.9 DDD/1000 outpatient-days during the assessment period (*p* < 0.001; Fig. 1H).

### Changes in patients’ costs on hospital stay, medication, antibiotics including the very-restricted antibiotics

**Fig 2:**
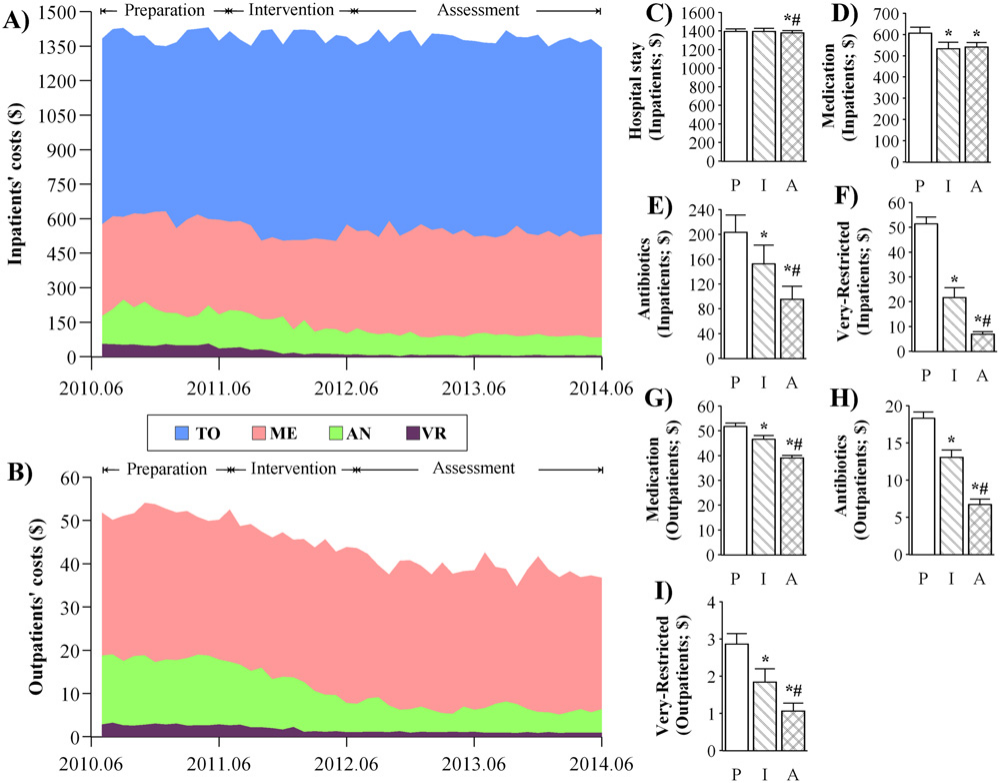
Changes in patients’ costs. The stack plots represent the average monthly data of costs for inpatients (A) and outpatients (B) during the preparation, intervention, and assessment periods. The costs are summarized as the costs on hospital stay (TO), medication (ME), antibiotics (AN), and very-restricted antibiotics (VR). Cross-sectional analyses were conducted by comparing the average yearly data on patents’ costs on hospital stay, medication, antibiotics, and very-restricted antibiotics (Very-Restricted) for both inpatients (C—F) and outpatients (G—I). P: preparation; I: intervention; A: assessment; *significant difference in intervention/assessment vs. preparation; ^#^significant difference in assessment vs. intervention.

The average patients’ costs during the 3 periods are shown in Fig. 2. In the inpatient settings, the total costs on hospital stay remained practically unchanged (*slope* = -0.71; *se* = -2.88; *p* = 0.804) during the intervention period, whereas the average costs on medication showed a significant decrease (*level* = -41.2; *se* = 17.7; *p* < 0.05) at the start of the intervention period (Table 2). The average costs on antibiotics decreased by $6.95 (*se* = 1.57; *p* < 0.001) per month during the intervention period for hospitalized patients (Table 2). Similarly, the costs for hospitalized patients on the very-restricted antibiotics showed a significant reduction (*slope* = -2.08; *se* = 0.42; *p* < 0.001) per month during the intervention period (Table 2). Cross-sectional analyses showed significant decreases in the average costs on hospital stay ($1396.2 vs. $1382.2; *p* = 0.041), medication ($606.7 vs. $541.8; *p* < 0.001), antibiotics ($203.7 vs. $95.4; a reduction of 53%; *p* < 0.001), and on the very-restricted antibiotics ($51.3 vs. $6.9; a reduction of 87%; *p* < 0.001) for hospitalized patients during the assessment period compared with the preparation period (Fig. 2C–2F).

**Table 2:** Regression analyses of ITS on the changes in patients’ costs.

	β1-trend (preparation)	β2-level change (intervention)	β3-trend change (intervention)	β4-level change (assessment)	β5-trend change (assessment)
**Inpatients’ costs**					
**TO**	0.62 (2.18)	-1.59 (21.66)	-0.71 (2.88)	0.60 (16.72)	-0.89 (2.05)
**ME**	-0.84 (1.90)	-41.22 (17.07)*	-3.59 (2.87)	43.84 (15.12)**	3.63 (1.82) *
**AN**	-2.06 (1.19)	15.95 (12.24)	-6.95 (1.57) ***	-1.82 (9.40)	8.02 (1.17)***
**VR**	-0.80 (0.32) *	-7.01 (3.39) *	-2.08 (0.42) ***	2.57 (2.53)	2.78 (0.32) ***
**Outpatients’ costs**					
**ME**	-0.11 (0.11)	-0.87 (1.13)	-0.49 (0.12) ***	-2.45 (0.77)**	0.46 (0.10) *
**AN**	-0.03 (0.08)	0.32 (0.77)	-0.81 (0.12) ***	-0.73 (0.67)	0.76 (0.09) ***
**VR**	-0.02 (0.01)	0.15 (0.16)	-0.14 (0.02) ***	0.31 (0.12) *	0.15 (0.01) ***

Changes in level and slope in patients’ costs on medications (ME), antibiotics (AN), and on the very-restricted (VR) antibiotics were analyzed for both inpatients and outpatients in the 65 general hospitals. Patients’ total costs during hospital stay (TO) were analyzed for inpatients. The parameters of β1 to β5, expressed as Mean (SE), were described in the Methods.

*p < 0.05;

** *p*< 0.01;

***p < 0.001.

In the outpatient settings (Fig. 2B), the average patients’ costs on medication, antibiotics, and on the very-restricted antibiotics decreased significantly by $0.49 (*se* = 0.12; *p* < 0.001), $0.81 (*se* = 0.12; *p* < 0.001), and $0.14 (*se* = 0.02; *p* < 0.001) per month during the intervention period, respectively (Table 2). Cross-sectional analyses showed significant decreases in the average outpatients’ costs on medication ($51.7 vs. $39.1; *p* < 0.001), antibiotics ($18.3 vs. $6.7; a reduction of 63%; *p* < 0.001), and on the very-restricted antibiotics ($2.9 vs. $1.1; a reduction of 62%; *p* < 0.001) during the assessment period compared with the preparation period (Fig. 2G–2I).

### Changes in hospital stay as well as duration of peri-operative antibiotic treatment in the 4 types of clean surgeries

**Fig 3:**
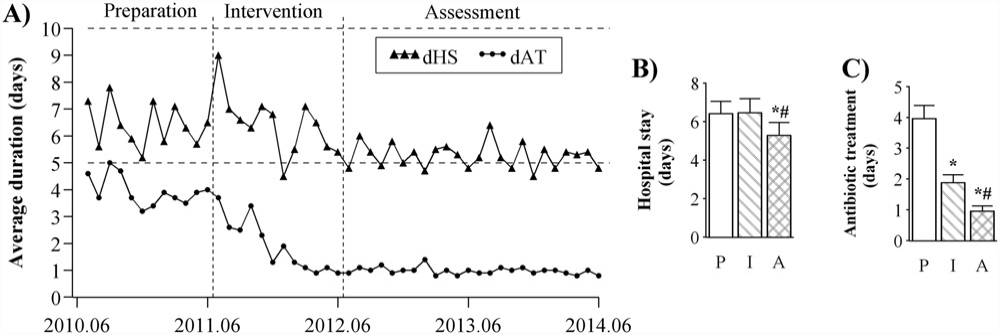
Changes in hospital, duration of peri-operative antibiotic treatment. Time series of duration of hospital stay (dHS) and peri-operative antibiotic treatment (dAT) for patients undergoing 4 types of clean procedures of thyroidectomy, breast, hernia, and orthopedic procedures were plotted (A). Cross-sectional analyses were conducted by comparing the duration of patients’ hospital stay (B) and the duration of peri-operative antibiotic treatment (C) received by the surgical patients during the 3 study periods. P: preparation; I: intervention; A: assessment; *significant difference in intervention/assessment vs. preparation; ^#^significant difference in assessment vs. intervention.

The average duration of hospital stay as well as peri-operative antibiotic treatment for patients receiving the 4 types of clean surgeries of thyroidectomy, breast surgery, hernia, or orthopedic procedures in the 65 general hospitals are shown in Fig. 3. The average duration of hospital stay decreased by 0.16 day (*se =* 0.03; *p* < 0.001) per month during the intervention period; the duration of peri-operative antibiotic treatment received by the surgical patients decreased by 0.19 day (*se =* 0.02; *p* < 0.001) per month during the intervention period (Table 3). Cross-sectional analyses showed significant decreases in the duration of hospital stay (6.41 vs. 5.27 day; *p* < 0.001) and the duration of peri-operative antibiotic treatment (3.97 vs. 0.96 day; *p* < 0.001) during the assessment period compared with the preparation period (Fig. 3B and 3C).

**Table 3:** Regression analyses of ITS on the changes in duration of hospital stay and peri-operative antibiotic treatment in the 4 types of clean surgeries.

	β1-trend (preparation)	β2-level change (intervention)	β3-trend change (intervention)	β4-level change (assessment)	β5-trend change (assessment)
**dHS**	-0.04 (0.03)	1.50 (0.34)	-0.16 (0.03) ***	0.11 (0.22)	0.19 (0.03) ***
**dAT**	-0.09 (0.02)***	0.30 (0.21)	-0.19 (0.02) ***	0.71 (0.14) ***	0.27 (0.02) ***

Changes in level and slope in the days of hospital stay (dHS), as well as the duration of peri-operative antibiotic treatment (dAT) received by patients undergoing 4 clean surgeries of thyroidectomy, breast surgery, hernia, or orthopedic procedures in the 65 hospitals were analyzed. The parameters of β1 to β5, expressed as Mean (SE), were described in the Methods.

*p < 0.05;

**p < 0.01;

***p < 0.001.

## Discussion

In summary, the impacts of a national action plan for antibiotic stewardship in China were evaluated with reference to the goals set by the Action Plan, including the frequency and intensity of antibiotic consumption as well as the duration of antibiotic prophylaxis in clean surgeries of thyroidectomy, breast, hernia, and orthopedic procedures. A total of 65 public general hospitals were included, and data were extracted from pharmacy modules of respective hospital information systems. Longitudinal analyses were conducted on the average monthly data by regression analysis of ITS; cross-sectional analyses were conducted on the average yearly data using the mixed model. Regression analyses of ITS revealed consistent and significant trend changes (*β3*) in the frequency and intensity of antibiotic consumption, the patients’ costs on antibiotics, and the duration of antibiotic treatment received by surgical patients undergoing the 4 clean procedures during the intervention period (Tables 1, 2, and 3). Cross-sectional analyses show that the antibiotic prescribing rates during the assessment period were reduced to 35.3% and 12.9% (Fig. 1D and 1E) in inpatient and outpatient settings respectively, indicating that the goals set by the Action Plan were achieved (60% for inpatients and 20% for outpatients as described in the Methods). During the preparation period, the average yearly intensity of antibiotic consumption was 76.6 DDD/100 bed-days (Fig. 1G) in inpatient settings, which was comparable to the previously reported data (80.1 DDD/100 bed-days) [5]. In contrast, the intensity was reduced to 35.9 DDD/100 bed-days (Fig. 1G) during the assessment period, which was lower than the goal set by the Action Plan (40 DDD/100 bed-days). Meanwhile, patients’ costs on antibiotics were reduced significantly by 53% (Fig. 2E) and 62% (Fig. 2H) in inpatient and outpatient settings during the assessment period compared with the preparation period, respectively. In effect, the duration of hospital stay were analyzed for all hospitalized patients as well, showing that there were no significant differences during the 3 periods investigated (data not shown); therefore, we selected a homogeneous subset of clean surgeries to evaluate the impacts of the Action Plan. The duration of peri-operative antibiotic treatment received by surgical patients undergoing the 4 types of clean procedures decreased to less than 24 hour (< 24 hour set by the Action Plan) during the assessment period, which was concomitant with a slight yet significant decrease in the duration of hospital stay (Fig. 3B and 3C).

The purpose of the Action Plan as a combination of professional and administrative strategies was to promote rational antibiotic utilization to contain the prevalence of antibiotic resistance and patients’ costs. The Action Plan focused on the managerial strategies. The effectiveness and efficiency of the managerial strategies can be attributed, at least partially, to the organizational structure in China’s public hospitals. The chief administrators are appointed jointly by higher healthcare administrations and cadre authorities, taking full responsibilities of day-to-day performance, including procurement of medicines and employment decisions [16]. Healthcare professionals working in public hospitals are subject to civil service rules as well as the supervision from cadre authorities [16], meaning that disciplinary actions caused by infringement on the antibiotic guidelines lead to the cessation of the prescribers’ medical career. In implementing the strategies initiated by the Action Plan, members of DTC and directors of all relevant departments hold weekly meetings, by which issues in antibiotic administration were identified and discussed and prescribers in question are interviewed. In routine practice, clinical pharmacists are conferred with more power to participate in daily ward-rounds and concurrent pharmacy validation. Furthermore, as a managerial tool, healthcare authorities conduct two supervision sessions each year, during which the hospitals’ performances relating to antibiotics are inspected. All the members of our research team agree that prescribers and clinical pharmacists are working under more pressure from the management and peers.

Since China’s private hospitals, which handled about 5% of total outpatient and inpatient services [13], are privately funded and operated for profit with more autonomy, the Action Plan of antibiotic stewardship concentrated on public hospitals, which are roughly divided into national, provincial, municipal, and county-level hospitals. Our pilot study on the national and county-level public hospitals showed larger variation in the collected data; therefore, they were excluded from this study to achieve statistical homogeneity. Though statistical analyses showed slight yet significant differences between secondary and tertiary hospitals, separate data analyses using hospital category as a factor reached the same conclusions as they were analyzed as a whole (data not shown). Besides, only 65 out of 300 hospitals were admitted in this study, which was mainly due to three reasons: (1) the lack of a fully-functional pharmacy management system in certain hospitals that were excluded from this study; (2) the concern for release of sensitive medical data; and (3) the stratified sampling method, which required a ratio of the number of tertiary hospitals to the number of secondary hospitals equaling about 9/10. A computerized pharmacy management system plays important roles in pharmacy validation and concurrent prescription monitoring with improved patient outcomes [17]. However, more than 50 hospitals that could not participate in this study lacked a functional pharmacy module in their hospital information systems during the study periods, especially in 2010. Although the avoidance of acquiring sensitive data, such as standardized mortality rates, was manifested in our research protocols, more than 100 of hospitals that refused to participate concerned the release of medical information on patients’ costs, resulting in the relatively lower response rate (152/300), which was confirmed in our follow-up calls. In addition, the impacts of the Action Plan on the duration of antibiotic treatment in clean surgeries were evaluated in only 4 types of clean surgeries of thyroidectomy, breast, hernia, and orthopedic procedures, since these 4 surgeries could be identified as clean procedures from the available data. We examined the duration of antibiotic treatment other than the duration of antibiotic prophylaxis, since whether the antibiotic treatment was for prophylactic purpose or for therapeutic purpose could not be identified from the collected data in the year of 2010. The compliance of administering antibiotic prophylaxis 30 to 120 minute prior to surgical incision was not evaluated quantitatively in this study, since recording the timing of prophylactic antibiotic administration electronically required technical issues of modifying the pharmacy management systems, which occurred differentially in the investigated hospitals.

Along with the antibiotic stewardship program in public hospitals, the Chinese government began to strengthen antibiotic utilization policy specific to primary healthcare institutions in the community and in rural clinics, which will be evaluated in our future studies. We hope that the fundamental changes prompted by the Action Plan can gradually turn into institutionalized and standardized clinical practice in all healthcare institutions so as to effectively address the global threat—antibiotic resistance.

## Conclusions

In conclusion, the antibiotic stewardship in China was effective in reducing the frequency and intensity of antibiotic use in public hospitals. Furthermore, segmented regression analysis of ITS reveals how these changes in antibiotic prescriptions changed in different settings in various types of hospitals, which will provide evidence for healthcare policy makers when for future policy interventions on antibiotic stewardship.
